# Surgical Therapy of Infective Prosthesis Endocarditis following TAVI: A Single Center’s Experience

**DOI:** 10.3390/diagnostics14121259

**Published:** 2024-06-14

**Authors:** Alexander Weymann, Ali Saad Merzah, Arian Arjomandi Rad, Lukman Amanov, Thanos Athanasiou, Bastian Schmack, Aron-Frederik Popov, Arjang Ruhparwar, Alina Zubarevich

**Affiliations:** 1 Department of Cardiothoracic, Transplant and Vascular Surgery, Hannover Medical School, 30625 Hannover, Germany; 2 Medical Sciences Division, University of Oxford, Oxford OX1 2JD, UK; 3 Department of Surgery and Cancer, Imperial College London, London SW7 2AZ, UK; t.athanasiou@imperial.ac.uk

**Keywords:** prosthesis endocarditis, TAVI, surgical aortic valve replacement, TAVI prosthesis endocarditis

## Abstract

Background/Objectives: Infective prosthesis endocarditis (IE) following transcatheter aortic valve implantation (TAVI) presents significant management challenges, marked by high mortality rates. This study reviews our center’s experience with surgical interventions for IE in patients post-TAVI, focusing on outcomes, challenges, and procedural complexities, and providing an overview of the limited literature surrounding this subject. Methods: This study was executed as a comprehensive retrospective analysis, targeting the clinical outcomes of surgical treatment in patients presenting with PVE following TAVI procedures at our institution. From July 2017 to July 2022, we identified five patients who had previously undergone transfemoral transcatheter aortic valve implantation and were later diagnosed with PVE needing surgery, strictly adhering to the modified Duke criteria. Results: All surgical procedures were reported successful with no intra- or postoperative mortality. Patients were predominantly male (80%), with an average age of 76 ± 8.6 years, presenting mostly with dyspnea (NYHA Class II). The mean follow-up was between 121 and 1973 days, with outcomes showing no occurrences of stroke, myocardial infarction, or major bleeding. One patient expired from unrelated causes 3.7 years post-surgery. The operative and postoperative protocols demonstrated effective disease management with enhanced survival and minimal complications. Conclusions: The surgical treatment of IE following TAVI, though challenging, can be successfully achieved with careful patient selection and a multidisciplinary approach. The favorable outcomes suggest that surgical intervention remains a viable option for managing this high-risk patient group. Our study also highlights the scarce literature available on this topic, suggesting an urgent need for more comprehensive research to enhance understanding and improve treatment strategies. Future studies with larger cohorts are needed to further validate these findings and refine surgical strategies for this growing patient population.

## 1. Introduction

The management of prosthesis infective endocarditis (IE) following transcatheter aortic valve implantation (TAVI) embodies a significant clinical challenge that demands a comprehensive, interdisciplinary approach and high-level medical expertise. While prosthesis IE remains a relatively infrequent complication, its occurrence ranges between 1% and 6% of cases, thereby underscoring its clinical significance [1]. The growing number of surgical and transcatheter valve replacements annually has contributed to the increased prevalence of prosthetic valve endocarditis (PVE) globally [1]. In a joint statement from STS and EACTS regarding aortic valve replacement in low-risk patients, the investigators reported that, over the past five years, TAVI explantation or generally surgery after TAVI for various indications has become the fastest-growing operation in the STS database [2]. Despite advancements in both antimicrobial and surgical therapies, the management of PVE following TAVI procedures remains complex and is associated with alarmingly high mortality rates, reported between 24% and 46% [3]. The uptake of TAVI has not only introduced a novel therapeutic solution for patients presenting with severe aortic stenosis, but it has also provided a unique subset of PVE cases, characterized by distinct morphological, microbiological, and clinical attributes [4,5].

Infective endocarditis following TAVI presents significant challenges for surgeons due to its high-risk profile and potential for severe complications such as stroke, bleeding, and sepsis. As TAVI indications expand to include lower-risk populations, untreated prosthesis endocarditis can lead to fatal outcomes. The surgical management of PVE in this setting carries substantial risks, including the development of paravalvular abscesses affecting the aortic root and aortomitral continuity, increased stroke risk, and tissue damage due to adhesions. These issues highlight the critical need for prompt and effective treatment strategies to reduce the high mortality and morbidity associated with this serious condition [6,7,8].

The aim of our study is to comprehensively review and analyze our center’s experience with the surgical treatment of active infective prosthesis endocarditis in patients who previously underwent a TAVI procedure. This study seeks to evaluate the outcomes, challenges, and complications encountered in the management of such cases, with a particular focus on the surgical treatment employed to address active PVE following TAVI. By examining our center’s approaches, patient outcomes, and the complexity of cases, we aim to provide valuable insights into the optimal strategies for managing this constantly growing high-risk patient population, enhancing our understanding of the procedural and postoperative challenges. Furthermore, we aim to explore the current limited body of evidence around this extremely high-risk population of patients.

## 2. Materials and Methods

### 2.1. Study Design and Participant Selection

This study was executed as a comprehensive retrospective analysis, targeting the clinical outcomes of surgical treatment in patients presenting with PVE following TAVI procedures at our institution. From July 2017 to July 2022, we identified 5 patients who had previously undergone transfemoral transcatheter aortic valve implantation either at our institution or elsewhere and were later diagnosed with PVE needing surgery, strictly adhering to the modified Duke criteria. The choice of a retrospective design allowed for an in-depth analysis of real-world outcomes, albeit with an acknowledgment of the limitations inherent to this approach, such as potential selection bias and the small sample size, which may affect the generalizability of our findings.

### 2.2. Preoperative Assessment

A comprehensive preoperative evaluation was systematically conducted for each patient by our institutional multidisciplinary Endocarditis Team, including experts in cardiac surgery, cardiac anesthesia, interventional cardiology, microbiology, and the Antibiotic Stewardship Team (AST). This collaborative effort ensured the formulation of a personalized treatment strategy, addressing the unique challenges posed by IE in patients following TAVI, thus optimizing patient care and treatment efficacy.

### 2.3. Data Collection and Ethical Considerations

Data were retrospectively gathered, encompassing demographics, clinical presentations, laboratory findings, echocardiographic and hemodynamic parameters, intraoperative variables, and postoperative outcomes. This information was extracted from electronic medical records, ensuring adherence to the highest ethical standards in line with the Declaration of Helsinki (2013 revision). Ethical approval was granted by the Medical School of Hannover’s Institutional Review Board (Nr. 11358_BO-K_2024). Informed written consent was obtained from all participants, guaranteeing the confidentiality and anonymity of their data throughout the analysis.

### 2.4. Operative Techniques

**Figure 1:**
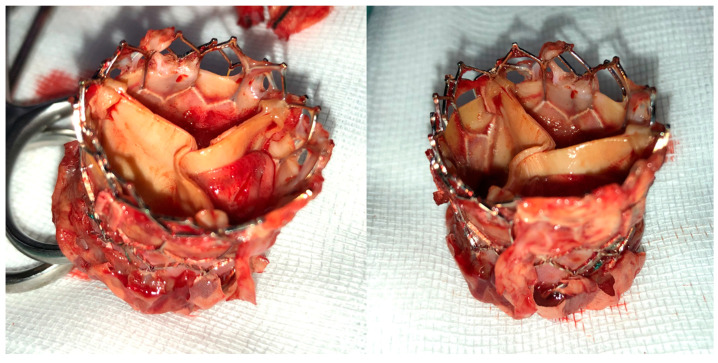
Intraoperative image of explanted TAVI prosthesis in a patient presenting with acute infective endocarditis following TAVI.

Access to the chest was obtained through a median sternotomy. During the surgical procedure, normothermic cardiac arrest was maintained to optimize myocardial protection. Cardiopulmonary bypass (CPB) was established via the direct cannulation of the ascending aorta and the right atrium. For myocardial protection, Custodiol HTK solution (Köhler Chemie GmbH, Bensheim, Germany) was employed, administered either through the aortic route or directly into the coronary ostia in the presence of a significant aortic regurgitation. All procedures were carried out under continuous CO_2_ insufflation. Following the careful disengagement of the TAVI prosthesis utilizing two forceps (Figure 1), the native aortic valve was excised completely in a regular for the surgical aortic valve replacement (SAVR) manner. This step was critical for ensuring the thorough removal of the diseased valve while minimizing the risk of damaging the adjacent cardiac structures such as parts of the conduction system, ventricular septum, and anterior mitral leaflet, thus highlighting the surgical precision and the emphasis on minimizing perioperative risk. In patients receiving the Perceval™ valve prosthesis (Corcym, Saluggia, Italy), the implantation began with three 4.0 Prolene™ guiding sutures at each nadir’s centroid, followed by the meticulous implantation of the Perceval sutureless aortic valve using the Snugger technique as described previously [9,10], ensuring its optimal positioning and stability within the aortic annulus. For patients who underwent a SAVR with a biological prosthesis, the procedure involved the careful placement of pledgeted annular sutures, followed by the implantation of the valve prosthesis, as described previously [11].

For the aortic root replacement using a biological conduit, the aortic sinuses were removed, the coronary buttons were prepared for re-implantation, and the 2.0 pledgeted annular sutures were placed. A biological conduit consisting of a 23 mm Perimount Magna Ease aortic valve (Edwards Lifesciences, Irvine, CA, USA) and a 28 mm Hemashield (Getinge, Sweden) tube prosthesis was constructed at the back table. After passing the annular sutures through the sewing ring of the valve prosthesis, the biological conduit was parachuted into the aortic annulus, and the sutures were tightened. The coronary buttons were carefully tailored and re-implanted into the prosthesis with 5.0 Prolene™ running sutures. The aortotomy closure was achieved by two 4.0 Prolene™ double-layered sutures, reinforced with pledgets, ensuring the structural integrity of the aortic wall and minimizing the potential for postoperative bleeding complications.

After completing the de-airing process to remove the air entrapped within cardiac chambers, thereby mitigating the risk of air embolism upon the cessation of CPB support, the valve function was meticulously evaluated via transesophageal echocardiography. The CPB support was discontinued, and anticoagulation was completely reversed to restore the hemostasis. The closure of the sternum was performed using stainless steel wires in a regular manner.

### 2.5. Postoperative Care Protocol

Postoperatively, a tailored 6-week regimen of intravenous antibiotics was administered based on the identified pathogens and antimicrobial susceptibility testing, as determined by our clinical experience with the surgical treatment of SAVR PVE and the multidisciplinary Endocarditis Team at our institution according to the current ESC endocarditis guidelines [12]. Prior to discharge, each patient’s valve functionality and hemodynamic status were evaluated via transthoracic echocardiography to ensure the success of the surgical treatment and to potentially guide further management.

### 2.6. Outcome Measures

The primary outcome measures were defined as the postoperative mortality rates at 30 days and 3 months and the operational success of the implanted device, assessed through transthoracic echocardiography. Secondary outcomes included the identification of postoperative complications, categorized according to the Valve Academic Research Consortium (VARC-2) standards [13].

### 2.7. Statistical Evaluation

Statistical analysis was conducted using IBM SPSS Version 28. The Shapiro–Wilk test assessed the normality of data distribution. Depending on this assessment, quantitative data were summarized as means ± standard deviation (SD) or medians with interquartile ranges (IQRs), and categorical data as frequencies and percentages.

## 3. Results

### 3.1. Baseline Data

Five (one female and four males) patients (mean age 76 ± 8.6 years) with acute aortic valve prosthesis endocarditis following transfemoral transcatheter aortic valve implantation (mean time post-TAVI 1.97 ± 0.76 years) underwent surgical treatment at our institution. The mean EuroSCORE II was 5.5 ± 1.5% with the mean EuroSCORE II of 2.27 ± 1.4% at TAVI. All patients presented with dyspnea NYHA Class II and preserved LVF (mean 58.4 ± 5.0%). The mean transaortic gradient prior to the surgical procedure was 9.0 ± 2.4 mmHg. Further details of patients’ baseline data are presented in Table 1.

### 3.2. Intraoperative Data

All patients underwent surgery via median sternotomy, and in all cases, the procedure was performed with an urgent indication. In four patients, the TAVI prosthesis could be explanted easily, so that an SAVR could be performed (a sutured prosthesis in two cases and a sutureless aortic valve prosthesis in the other two cases), and in one case, a full root replacement with the replacement of the ascending aorta had to be performed. The mean operating time was 194 ± 131.4 min, and the mean cross-clamp time was 65.2 ± 40.2 min. In three of the five cases, there was no pathogen detected on the explanted valve prosthesis (Table 2).

### 3.3. Postoperative Outcomes

All procedures were reported as successful with no intra- or perioperative mortality. There were no postoperative complications such as stroke, myocardial infarction, or bleeding in our cohort. All patients had an uneventful postoperative course with a short time at the ICU. The mean follow-up time was 756 ± 853 days. Four patients reported being in a good clinical state at the time of follow-up; one patient died of cancer 3.7 years after the surgery. Other postoperative surgical-related outcomes are presented in Table 3.

## 4. Discussion

Infective endocarditis following TAVI is a complex and growing challenge both for cardiologists and cardiothoracic surgeons. With the expansion of TAVI indication into the mid- and low-risk group presented in the current ESC guidelines, the medical community is facing a growing number of patients presenting with PVE following TAVI [14]. This most feared complication is not uncommon, with a reported incidence of up to 1.6% per year and up to 40–70% mortality rate [15,16,17,18]. Although in over 80% of patients presenting with PVE after TAVI, the indication for reperforming SAVR is indisputable, only 2–14% of patients undergo a surgical procedure [19,20,21,22]. The surgical community’s reluctance to treat TAVI PVE aggressively stems from several factors. There is a pervasive fear of high mortality rates linked to the complex additional surgical interventions required. Moreover, patients often present with poor medical conditions due to sepsis and other comorbidities. Additionally, there is a lack of experience in managing such high-risk cases, compounded by limited research data on surgical therapy for TAVI PVE. Most of the available data derive from smaller cohort studies and individual case reports, further complicating decision-making processes.

Although it has been reported in the literature that the occurrence of PVE after SAVR and TAVI is similar, there are several factors contributing to the unique challenges associated with TAVI-related PVE compared to PVE following SAVR, which are indicated below.

### 4.1. Microbiological Profile

TAVI-related IE can involve different bacterial strains compared to SAVR bioprosthetic valve endocarditis [23]. This necessitates a high index of suspicion for IE in patients with TAVI who present with signs of infection. As most of the cases of TAVI PVE we are dealing with at the moment are coming from the intermediate- and high-risk cohorts (prior to the extension of the TAVI indications into the low-risk group), we are looking at the pathogen spectrum of elderly patients, with streptococci and staphylococci being the most common pathogens reported in the literature, in line with our cohort (Table 1). These pathogens have been reported to be isolated in over 30% of the PVE cases. Interestingly, unlike the pathogen spectrum of patients presenting with PVE following SAVR, in over 25% of TAVI PVE cases, enterococcus faecalis have been isolated [19,22,24,25,26,27]. Moreover, unlike the IE following SAVR, culture-negative TVAI PVE is rather uncommon (<5%) [28].

### 4.2. Anatomical Considerations

The specific design and implantation technique of TAVI valves can create a niche for bacterial colonization, particularly around the valve struts and anchoring mechanisms [23]. This can complicate the complete eradication of the infection during surgery. It has been reported that, in up to 40% of TAVI cases, paravalvular leakage (PVL) could be identified. Not only is PVL a predictive factor of short- and long-term mortality after TAVI, with a negative impact on the coronary flow, but it also significantly affects the incidence of PVE [29,30].

### 4.3. Patients’ Profile

TAVI recipients tend to be significantly older than SAVR patients and have multiple comorbidities, which can increase the risk associated with surgical intervention for PVE in the initial TAVI cohorts. According to several reports in the literature, early mortality after the surgical treatment of TAVI PVE depends on the initial risk profile and comorbidities of the patient at the time of the TAVI procedure. Therefore, as TAVI patients are getting significantly younger with the new guidelines for TAVI procedures, the early mortality should be decreasing compared to the previous high-risk TAVI cohorts [19,24,31]. Local complications related to the presence of the periannular abscess or the involvement of the mitral valve or aortomitral curtain are also not uncommon in patients with TAVI PVE referred for surgery. These patients tend to carry higher operative risk and often show higher perioperative mortality and morbidity than those without an abscess. The current body of literature suggests that up to 25% of patients presenting with TAVI PVE have a periannular abscess and need aortic valve retrieval and replacement, as well as an aortic root replacement, thus carrying higher operative risk. Luckily, in our cohort, we did not observe any cases of periannular abscess, but we still had to perform aortic root replacement on patient #1 due to the severe adhesions of the TAVI prosthesis to the aortic annulus (Table 2). Regardless of the surgical procedure, none of our patients suffered any postoperative complications.

### 4.4. Surgical Management and Outcomes

Our study demonstrates the feasibility and potential benefits of surgical intervention for carefully selected TAVI patients with PVE. The meticulous surgical approach described, emphasizing the radical removal of the infected TAVI prosthesis and implantation of a new valve, aligns with established surgical techniques for PVE [32].

Importantly, every patient in our investigation realized a successful outcome, defined by the absence of mortality at thirty days and three months, valve-related complications, stroke, myocardial infarction, and appropriate valve function at discharge assessed through echocardiographic evaluation (Table 3).

Although the current literature fails to demonstrate significantly better outcomes of surgery compared to conservative therapy in patients with TAVI PVE presenting with a high-risk profile [33,34], the most current data from the mid- and low-risk cohorts suggest that the outcomes after surgery tend to mimic those of patients presenting with PVE following SAVR [35,36].

Our findings on mortality rates (100% survival at 30 days and 3 months) are consistent with the range reported in the literature for surgical management of PVE (20–50%) [37,38,39]. Variations in mortality likely reflect differences in patient demographics, the severity of the disease, the involvement of the aortic root, and the surgical experience of the operating team. Additionally, the timing of surgery may influence outcomes, with earlier radical surgery, especially in patients with larger vegetations and/or the involvement of the aortic annulus, potentially leading to better outcomes.

While, due to the small sample size, our study might not be significantly representative regarding complication rates, existing research highlights the high-risk nature of surgery for PVE following TAVI. Potential complications include paravalvular abscess formation, stroke, and bleeding, none of which were found in our cohort [23]. Differences in reported complication rates across studies may be attributed to variations in patients’ clinical characteristics, the surgical techniques employed, and postoperative care protocols [40,41,42].

### 4.5. Implications for Clinical Practice

This study informs clinical decision-making in several ways, including the following:

Refined patient selection: Our findings highlight the necessity for a risk stratification tool to enhance patient selection processes for TAVI-related PVE. Patients who are carefully selected can greatly benefit from early and radical surgical interventions. The potential use of sutureless valve prostheses (Perceval, Corcym, Saluggia, Italy) simplifies the surgical procedure and reduces operating and cross-clamp times (Table 2), as previously reported in our experience with patients presenting with acute IE [43]. A nuanced understanding of the patient’s clinical profile, including age, comorbidities, and the severity of PVE, is crucial in determining surgical candidacy and the timing of the procedure.

Importance of multidisciplinary care: The success of our cases emphasizes the importance of the multidisciplinary Endocarditis Team, involving cardiologists, cardiac surgeons, infectious disease specialists, and other healthcare professionals, to optimize patient care throughout the management course [44,45].

Tailored postoperative care: Meticulous postoperative care, including targeted antibiotic therapy and close monitoring for complications, is paramount for optimizing postoperative outcomes. In our center, all patients presenting with PVE are treated with intravenous antibiotics for six weeks according to the pathogen spectrum in blood and on the diseased explanted valve prosthesis (Table 3).

### 4.6. Future Prospects

Investigating and refining surgical techniques specifically designed for TAVI valve retrieval and reimplantation may further improve outcomes in this challenging patient population. This could involve exploring minimally invasive approaches or techniques that minimize the manipulation of the infected tissue, such as sutureless aortic valve prostheses [43].

With advancements in surgical technology, the trend toward minimally invasive surgical access is indisputable. Future studies could explore the potential role of minimally invasive surgery in the management of TAVI-related PVE, particularly for high-risk surgical candidates ineligible for sternotomy [46].

While this study highlights the clinical challenges of TAVI-related PVE, the underlying mechanisms of bacterial colonization and infection specific to TAVI valves remain incompletely understood. Some studies suggest that groin access in the majority of TAVI cases contributes to the fact and that one of the most common pathogens in patients with PVE following the TAVI procedure is *E. faecalis*, the growth of which is encouraged by the warm humid environment of the groin area [47]. Further research is needed to elucidate the precise factors that contribute to the development of PVE following TAVI procedures. This knowledge could inform the development of targeted preventive strategies and potentially novel therapeutic approaches.

### 4.7. Strengths and Limitations

The key strength of this study lies in its specific focus on TAVI-related PVE, which has become a growing concern with the expanding application of TAVI procedures throughout the whole risk spectrum of patients presenting with aortic valve stenosis. This focus offers valuable insights into the unique challenges and considerations for surgical management in this challenging patient population.

However, the limited sample size restricts the generalizability of the findings and precludes definitive conclusions on specific outcomes like complication rates and long-term survival. In addition to that, the retrospective nature of the study introduces potential biases in patient selection and data collection.

## 5. Conclusions

In the growing TAVI era and the extension of TAVI indications into the low-risk patients’ cohort, we are facing a rapidly growing number of patients returning for surgery due to prosthetic valve endocarditis following TAVI. Initial hesitancy from cardiothoracic surgeons toward these patients due to fear of presumably high mortality and perioperative complications should be reconsidered. Indeed, growing evidence of successfully performed procedures exists, providing a definitive treatment option for patients presenting with TAVI PVE. The promising results portrayed by the current literature as well as in this study could encourage further consideration not only to provide patients with adequate treatment but also to implement modern technologies such as sutureless valve prostheses or minimally invasive access in such patients.

## Figures and Tables

**Table 1:** Baseline data.

Variable	Patient #1	Patient #2	Patient #3	Patient #4	Patient #5
Age, years	64	72	84	84	78
Gender, female	no	no	yes	no	no
Risk factors and comorbidities					
COPD	no	no	no	no	no
Hypertension	yes	yes	yes	yes	yes
IDDM	no	yes	no	no	no
Hyperlipidemia	yes	yes	yes	yes	yes
Impaired kidney function	yes	no	yes	no	no
Dialysis	no	no	no	no	no
Malignancy	no	yes	no	no	no
NYHA Class	II	II	II	II	II
LV-EF, %	54	65	60	60	53
EuroSCORE II at TAVI, %	2.17	4.65	1.35	1.71	1.47
Time since the TAVI, y	1.25	3.2	2.15	1.81	1.46
TAVI prosthesis	Sapien 29 mm	CV Evolute R 26 mm	Sapien 26 mm	Sapien ultra 26 mm	Sapien ultra 29 mm
Prosthesis endocarditis	yes	yes	yes	yes	yes
Pathogen	*E. faecalis*	*Staph. epidermidis*	*Streptococcus gordonii*/*Staphylococcus hominis*	*Strep. salivarius*	*E. faecalis*
Antibiotic therapy	Linezolid/Piperacillin/Tazobactam	Rifampicin/Vancomycin	Ampicillin/Sulbactam	Ampicillin	Ampicillin/Gentamicin
Vegetation on the prosthesis > 1 cm	no	yes	no	yes	yes
Paravalvular leakage	no	no	yes	no	no
Paravalvular abscess	no	no	no	no	no
Embolic stroke	no	no	no	no	no
CRP, mg/L	10.2	17.9	2.4	8.4	2.1
Leukocytes, tsd/mcl	7.5	6.7	6	9.1	11.5
Creatinine, mcmol/L	168	105	101	92	1.2
GFR	37	62	45	66	58
Preoperative EuroSCORE II, %	7.77	5.05	5.04	3.7	5.9

COPD—chronic obstructive pulmonary disease, CRP—C-reactive protein, GFR—glomerular filtration rate, IDDM—insulin-dependent diabetes mellitus; LV-EF—left ventricular ejection fraction, NYHA—New York Heart Association, TAVI—transcatheter aortic valve implantation.

**Table 2:** Intraoperative data.

Variable	Patient #1	Patient #2	Patient #3	Patient #4	Patient #5
Urgent procedure	yes	yes	yes	yes	yes
Time since TAVI, years	1.25	3.2	2.15	1.81	1.46
Surgical procedure	Root replacement 23 mm Perimount Magna Ease, 28 mm HS	SAVR 23 mm Perimount Magna Ease	SAVR Perceval L	SAVR Perceval L	SAVR 29 mm Perimount Magna Ease
Operating time, min	425	169	101	139	136
Cross clamp time min	129	77	25	44	51
CPB time, min	223	112	39	65	70
Surgical access	sternotomy	sternotomy	sternotomy	sternotomy	sternotomy
Intraoperative blood transfusion, units	3	4	3	4	2
Pathogen on the valve prosthesis	*E. faecalis*	*Staph. epidermidis*	-	-	-

CPB—cardiopulmonary bypass; SAVR—surgical aortic valve replacement; TAVI—transcatheter aortic valve implantation.

**Table 3:** Postoperative outcomes.

Variable	Patient #1	Patient #2	Patient #3	Patient #4	Patient #5
ICU time, days	2	1	1	1	2
Time on ventilator, hours	17	8	11	15	11
In-hospital stay, days	14	8	7	13	6
Myocardial infarction	no	no	no	no	no
Maximal CK-MB, mmol/L	46	40	32	48	31
Stroke	no	no	no	no	no
Exploration for bleeding	no	no	no	no	no
Postoperative antibiotics	Ampicillin/ Gentamicin	Rifampicin/Vancomycin/ Gentamicin	Ampicillin/ Sulbactam	Ampicillin	Vancomycin/ Gentamicin
Follow-up time, days	1973	1344	197	144	121
Death at follow-up	no	Yes	no	no	no
Cause of death	-	malignoma	-	-	-
30-day mortality	no	no	no	no	no
3-month mortality	no	no	no	no	no
Clinical state at follow-up	good	-	good	good	good
NYHA Class	I–II	-	II	II	II

ICU—intensive care unit.

## Data Availability

The data are available from the corresponding author upon reasonable request.
